# Testing positive for Human Papillomavirus (HPV) at primary HPV cervical screening: A qualitative exploration of women’s information needs and preferences for communication of results

**DOI:** 10.1016/j.pmedr.2021.101529

**Published:** 2021-08-19

**Authors:** Sophie Mulcahy Symmons, Jo Waller, Emily McBride

**Affiliations:** a Department of Primary Care and Population Health, Institute of Epidemiology and Health Care, University College London (UCL), UK; b Centre for Interdisciplinary Research Education and Innovation in Health Systems (IRIS), School of Nursing, Midwifery and Health Systems, University College Dublin (UCD), Ireland; c Cancer Prevention Group, School of Cancer and Pharmaceutical Sciences, King’s College London (KCL), London, UK; d Department of Behavioural Science and Health, Institute of Epidemiology and Health Care, University College London (UCL), UK

**Keywords:** Human Papillomavirus, HPV, Cervical screening, Cancer, Education, Communication, Information needs, Qualitative

## Abstract

Human Papillomavirus (HPV) primary cervical screening was implemented across England during 2019, where cervical cell samples are first tested for HPV and cytology is used to triage HPV-positive results. Around 8.5% of women who attend test HPV-positive with normal cytology (HPV+/normal). We aimed to explore women’s information needs and suggestions for improvements to result communication following an HPV+/normal result, among those with higher and lower levels of education. In‐depth interviews were conducted with 30 women aged 24–63 who had tested HPV+/normal at routine screening. Secondary qualitative data, not previously reported, were analysed using Framework Analysis to compare themes between those with education lower-than-degree-level vs. degree-level-or-higher (n = 15 in each group). Regardless of education level, women had unanswered questions about their result meaning and the HPV primary screening protocol. Expectations of cervical screening did not always match the service provided, especially regarding content of letters and mode of result delivery. Women with lower education were less clear about the meaning of normal cytology and its link to HPV; and had difficulty sourcing information after their result. Pragmatic suggestions were made for preferences in content, wording, format, and delivery of information in patient communications. Overall, our findings point to areas which can be used by policymakers and healthcare professionals to inform content and communication of results, as HPV primary screening continues to be implemented and refined worldwide. Future research should use these suggestions to develop patient materials and then test them to assess content engagement and information recall.

## 1 Introduction

Human Papillomavirus (HPV) is a sexually transmitted infection responsible for nearly all cervical cancers (Jemal et al., 2010). Around 8 in 10 women are infected with HPV at least once in their lifetime (Chesson et al., 2014). In England, the National Health Service Cervical Screening Programme (NHSCSP) recently implemented routine HPV primary screening, where cervical cell samples are first tested for HPV and cytology is used to triage HPV-positive results, which is comparable to other HPV primary programmes internationally (Anttila et al., 2015, World Health Organisation, 2013, Huh et al., 2015). HPV primary screening is predicted to prevent up to 563 cervical cancers each year in the United Kingdom (Castanon et al., 2017). Under HPV primary screening in England, women are invited to be screened every 3 or 5 years, depending on age. Around 8.5% of women are expected to test positive for HPV with normal cytology (HPV+/normal) each year; a result which is specific to the HPV primary screening algorithm, carrying a low absolute cancer risk (Rebolj et al., 2019). Women who test HPV+/normal are recalled early to screening at 12 months to test for persistent HPV infection; and can receive this result three consecutive times before referral to colposcopy (Public Health England, 2017).

Concerns have been expressed regarding how accurately women will interpret the meaning of an HPV+/normal result, partly due to lack of immediate follow-up or routine clinical contact. Some women may be learning about the link between HPV, its sexually transmitted nature, and cervical cancer for the first time (McBride et al., 2020a). A recent content analysis of free-text survey answers among women attending HPV primary screening in England found that attempts to understand results was one of the most common themes. Around half of women testing HPV+/normal (52%) recorded questions about the meaning of HPV and cervical cancer (Marlow et al., 2020). Wider systematic review and research findings have also indicated that low knowledge and understanding of an HPV-positive result may be linked to adverse emotional responses, such as anxiety and sexual distress (McBride et al., 2020b, McBride et al., 2020c, McBride et al., 2021).

Although existing literature has identified common themes following receipt of an HPV-positive result (Bennett et al., 2019, McBride et al., 2020c, Patel et al., 2018), little research has focused on ways to improve result communication. A mixed-method study in the UK assessed how women interpreted an information leaflet about cervical screening. They found that interpretation difficulties were common, especially for women with lower education, lower numeracy, and an ethnicity other than white (Okan et al., 2019). Improving patient communication materials and reducing inequalities in burden therefore remain key priorities at cervical screening. However, to date, no research has explored pragmatic strategies for HPV result communication within a routine cervical screening programme. Further, little is known about distinct information needs of women receiving an HPV+/normal result at HPV primary screening; especially in those from lower educational backgrounds, where health literacy may be lower.

The aim of this secondary qualitative analysis was to explore women’s information needs and their suggestions for improvements to result communication following an HPV+/normal result at routine HPV primary screening in England. We also compared themes between women with lower than degree level vs. degree level or higher education to assess whether information needs differed based on educational attainment.

## 2 Methods

We performed secondary thematic analysis using interview data from a primary qualitative study exploring reasons for anxiety in women testing HPV+/normal (McBride et al., 2020a). The data used in this study had not been analysed in the primary study. Women aged 24–63 who had received an HPV+/normal result were recruited from two NHSCSP HPV primary screening sites in England (North West London and Greater Manchester). Supplementary File 1 contains the NHSCSP result letter women received as part of the English HPV primary screening pilot. In the primary qualitative study, women were purposively sampled based on survey answers to represent varying anxiety scores and demographic characteristics (education, age, ethnicity). Further details have been published elsewhere (McBride et al., 2020a). The semi-structured interviews followed a topic guide incorporating women’s reactions to result letters, information needs, and suggestions for improvement (see Questions 3, 4, 9, 14–16 in Supplementary File 2). Face-to-face interviews were carried out by EM between 28/06/19 and 31/08/19, audio-recorded, and transcribed verbatim.

Data were coded using qualitative analysis software NVivo 12 (QSR International Pty Ltd., 2020). SMS and EM read all transcripts; and SMS developed the initial codes and preliminary framework. The codes were discussed and refined iteratively until there was consensus on the final thematic framework. SMS coded all transcripts and EM independently coded 10% of the transcripts (n = 3), which indicated good inter-rater reliability (Kappa = 0.72). Once all data had been coded, it was summarised in a framework matrix to compare themes between women with lower-than-degree (O-level, ONC/BTEC, higher education qualification below degree) vs*.* degree-level-or-higher education. Education categorisation was chosen for consistency with previous HPV research (McBride et al., 2020b, Kola-Palmer and Dhingra, 2020) and equal distribution of numbers (n = 15 in each group). Framework analysis was performed to facilitate comparisons both within and between cases (Gale et al., 2013).

Ethical approval was granted from Health Research Authority Research Ethics Committee (18/EM/0227), Confidentiality Advisory Group (18/CAG/0118), and Cervical Screening Research Advisory Committee (ODR1819_005).

## 3 Results

Interviews were conducted with 30 women: 15 with lower-than-degree education (LE) and 15 with degree-or-higher education (HE). Women attended the interview on average 35.5 days after their HPV+/normal result (range: 22–76 days). Table 1 displays participant characteristics by education.

**Table 1:** Descriptive characteristics overall and by low vs. high education group.

	**Overall (N = 30)**	**Lower than Degree education (N = 15)**	**Degree or higher education (N = 15)**
***Highest educational qualification***			
University Degree or higher	–	–	15
Higher education qual. (below Degree)	–	7	–
Upper secondary (A-level)	–	1	–
Vocational qualification (ONC/BTEC)	–	2	–
Lower secondary (O-level)	–	5	–
**Age** (Median, Range)	36 (24–63)	42 (25–62)	34 (24–63)
***Ethnicity***			
White	22	13	9
Black/Mixed/Asian	8	2	6

***Relationship status***±			
No current partner	7	4	3
Current partner	23	11	12

***NHS site***			
Manchester	20	11	9
London	10	4	6

***HPV+/normal result***			
1st result	21	10	11
2nd or 3rd result*	9	5	4

*Abbreviations:* HPV = human papillomavirus. A-level = Advanced Level General Certificate of Education. ONC/BTEC = Ordinary National Certificate/Business and Technology Education Council. O-level = Ordinary level General Certificate of Education.

± Relationship status refers to self-report of a current partner, which may or may not be sexual in nature though is likely to be for the majority.

* Women had tested HPV+/normal for a 2nd or 3rd consecutive time at their 12‐month recall at HPV primary screening.

**Fig. 1:**
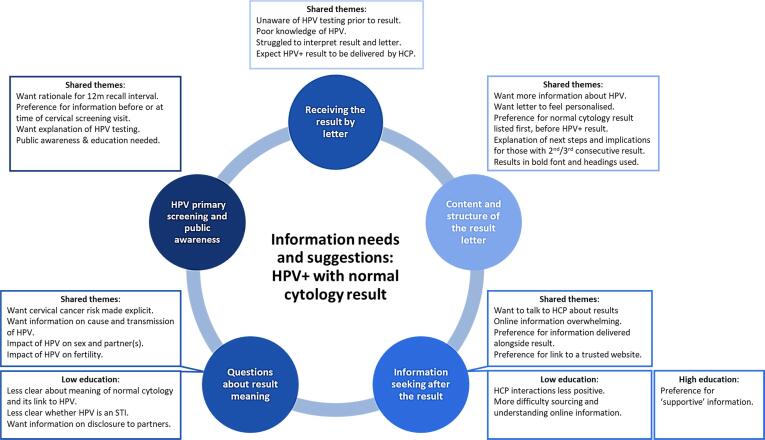
Thematic summary of results; information needs overall and between low and high education groups.

Women’s experiences, information needs, and suggestions covered five themes: (i) receiving the result by letter; (ii) content and structure of the result letter; (iii) information seeking after the result; (iv) questions about result meaning; and (v) HPV primary screening and public awareness. Overall, information needs were relatively consistent across high and low education groups, but nuanced differences were identified and are highlighted where applicable. Fig. 1 presents an overview of the thematic findings.

### 3.1 Receiving the result by letter

Most women were unaware that they could test HPV-positive at cervical screening until after they received their result, except a minority who had been informed at their screening appointment. Several reported confusion regarding the information in their result letter and could only accurately recall the HPV-positive aspect. Women with lower education particularly struggled to recall their normal cytology result.

*“I only recognised that there was another test or there’s another result for cell something, erm, when I was doing the survey for this [study]… I probably didn’t pay attention to it because it just said ‘normal’.”* (P6, LE)

Regardless of education level, for some, there was a prior expectation that they would only receive a mailed letter if their result was negative and “*good news*”, whereas “*if it was bad news I’d hear on a phone call or something” (P24, HE).* Others expected to receive a follow-up call or appointment from a healthcare professional (HCP):

*“It might have been a lot better if the doctor… someone from the practice phones, could phone me and say, “We’ve received this, and how do you feel about it?”* (P26, HE)

### 3.2 Content and structure of result letter

Women had mixed views about the wording of the result letter. Some thought it was “*polite*” and “*well written*”. Others thought it was “*blunt*” and “*not very humanised*”. Many felt the letter was “*informative to a point*” but there was not enough information to understand the result. Several expressed that more information would have helped put their “*mind at rest*”. A few referred to an analogy they had read which helped them to understand HPV clearance by the immune system and reassured them that HPV+/normal was low risk:

*I think explaining it with an analogy like it’s… you know, it’s like a cold, your immune system can clear it, that… I thought that was quite clear.* (P18, HE)

Nearly all women wanted their letter to feel more personalised. Notably, women who had received a 2nd or 3rd consecutive HPV+/normal result after 12-month recall expressed a need for information in their letter covering the future screening protocol and treatment procedures:

*“The [same] standard letter is not appropriate and should be changed to standard letter one, standard letter two, standard letter three … and what action needs to be taken.”* (P28, HE)

Regarding letter structure, some said they would prefer to get the “*good news*” (i.e. normal cytology result) before the “*bad news*” (i.e. HPV-positive result). Some suggested the letter should be restructured to draw more attention to the HPV and cytology result separately. Pragmatic suggestions included highlighting results in bold; splitting results under subheadings for ‘cytology’ and ‘HPV’; and displaying key information as bullet points. A few suggested visual aids or signposting to an online video.

### 3.3 Information seeking after the result

Nearly all women searched online for information immediately after reading their letter. In some instances, online information was viewed as helpful. However, many found it unhelpful comparing the process to searching through a “*minefield” (P1, LE).* Women with lower education expressed difficulty sourcing and interpreting online HPV content. Often this was due to uncertainty about what information to trust and through reading conflicting information.

*“I don’t always like Googling things because there’s so much out there that how do you - how do you know what - what’s the right information and what’s not”* (P10, LE)

In contrast, women with higher education reported higher satisfaction and discussed the importance of supportive information sources. Some particularly liked the Jo’s Cervical Cancer Trust website (UK charity), describing it as on *“the emotional side… It was informative in a way that I could take what knowledge I could from it but not, it wasn’t so like black and white*” (P12, HE).

Some women visited a HCP after receiving their result for reassurance and clarification. Interactions with HCPs were reported as positive overall. However, some women with lower education reported less satisfaction from clinical interactions.

*“I kinda felt that she [nurse] didn’t really have any more information than what was already out there anyway… I guess I was just hoping to get a bit more reassurance really”* (P23, LE)

Some reported not visiting their GP so as not to “*waste their [HCP’s] time” (P24, HE).* Alternative suggestions, considered to be acceptable, included visiting a sexual health clinic or calling a helpline. A preference for additional information placed alongside the result was expressed by most women, such as in a leaflet. The majority said information should be given to them rather than “*the onus*” being on them to seek it out. Women expressed interest in receiving a link to “*a trusted website*” *(P15, LE).* Few reported reading the cervical screening information leaflet which was posted alongside their screening invitation letter.

### 3.4 Questions about result meaning

Nearly all women had unanswered questions relating to their HPV+/normal result. Topics suggested for inclusion in letters primarily centred on HPV-positivity and its link to cervical cancer. Information which women thought would help their interpretation included whether they had a high-risk HPV type, their short and long-term cancer risk, and cancer survival statistics. Several wanted information about sexual transmission, how they could test positive for HPV in a long-lasting relationship, and potential implications for their partners and risk of re-infection. Women with lower education expressed greater uncertainty about whether HPV was sexually transmitted and wanted this confirmed; as well as information on whether they should inform (ex)partners. A few younger women requested information on whether HPV could affect fertility.

Some wanted information about the normal cytology aspect of their HPV+/normal result, but this was a minor theme. Women with lower education expressed less clarity regarding how HPV and cytology results were related to one another; and were confused about how they could have “*HPV but [their] cervix was clear*” *(P4, LE)*. Some thought HPV and cytology results were *“completely separate*” *(P21, LE).*

### 3.5 HPV primary screening and Public awareness

Few women had heard of HPV primary screening at the time of the interview. Of those that had, most found out after their result and wanted the switch in methods made explicit earlier in the screening process. Regardless of whether women formally knew about HPV primary screening, nearly all commented on 12-month early recall to screening and wanted information to explain the rationale for the change in interval. Many expressed the need for better communication and information provision before or during their screen. Some wanted their screening nurse to explain HPV testing and its implications, to reassure and prepare them for a potential HPV-positive result.

*“I think what would have helped as well, the fact that if they [sample taker] had mentioned that this is a new test.”* (P7, HE)

*“I think if it was explained why you have to wait twelve months, it would be a lot better. A lot of things can happen in twelve months”* (P4, LE)

Minor themes included the lack of public awareness campaigns about HPV primary screening and the need for better education, particularly related to sexual health and the benefits of cancer screening.

## 9 Discussion

Our findings provide insights into the information needs of women receiving HPV+/normal results at routine HPV primary screening. We identified themes that build on, and contextualise, current literature on HPV primary screening knowledge and women’s experiences of receiving HPV-positive results. Regardless of education level, most women reported poor knowledge of HPV and HPV testing and had several unanswered questions about the meaning of their HPV+/normal result. Expectations of the cervical screening programme did not always match with the service provided, especially regarding content covered in letters and mode of result delivery. Pragmatic suggestions for improvements to routine information can be used to enhance patient communications and inform patient aspects of clinician training, as HPV primary screening continues to be refined and implemented worldwide.

Consistent with previous research on abnormal cervical screening results, most women struggled to interpret the meaning of the new HPV+/normal result (McBride et al., 2020c, Bennett et al., 2019). They expressed lack of clarity around the implications of HPV and wanted more information alongside their result, rather than having to seek it out themselves. Most women raised the need for clear information explaining their result meaning and addressing unanswered questions. Overarching suggestions for the content to be covered in the HPV+/normal letter included: the cause and transmission of HPV; risk of cervical cancer; rationale for screening follow-up and (lack of) treatment procedures; and impact of HPV on fertility and sexual partners. These findings corroborate a recent content analysis using survey data on information needs at HPV primary screening in England, which found that around half of women testing HPV+/normal recorded similar questions (Marlow et al., 2020). HPV self-sampling has been introduced recently in some countries targeted at women who have not attended routine screening and has been found to be acceptable to women regardless of demographic characteristics (Nishimura et al., 2021). Women offered self-sampling have expressed similar concerns to those attending routine screening, but with additional concerns relating to the accuracy of the self-sampling test (e.g. Tiro et al., 2019, Nishimura, 2021).

Key pieces of information reported as helpful, partly in this study and from other work internationally, includes that HPV is common; it can remain dormant for a long time; only long-lasting infection leads to cervical cancer; there are minimal effects on partners; and HPV is usually cleared by the immune system without needing treatment (Waller et al., 2005, Dodd et al., 2019). Novel to this study, we identified specific questions about the HPV+/normal result. Participants wanted to understand: the rationale behind the 12-month recall interval; the change to HPV primary screening methods; and the relationship between HPV and cytology. In addition, women receiving a second or third consecutive HPV+/normal result required different information, e.g.*,* differences in cancer risk and the need for colposcopy after three consecutive results made clear.

In terms of letter format and structure, women suggested that results could be listed in bold font with information split under separate headings, clearly distinguishing between different aspects (i.e., ‘HPV’ and ‘normal cytology’). Some also wanted the “good news” before the “bad news”, i.e., their normal cytology result first. These suggestions could be cost-effectively implemented in patient communications for most routine screening programmes. However, it is possible that ordering normal cytology information first may mean that some women overlook the HPV-positive aspect, if they do not read the full letter. Hence, HPV+/normal letter restructuring should be formally tested through research to assess impact on information recall and content engagement.

Most women went online or visited a GP to find information about testing HPV+/normal after their result. Searching online was largely reported as confusing and unhelpful, especially by women with lower-than-degree-level education. Other studies have found that women with lower education have lower HPV knowledge and difficulty interpreting key aspects of screening (Okan et al., 2019). This emphasises the need for accessible targeted information at the point of delivery (i.e., alongside results) from trusted health authorities and/or signposting in letters to official websites. Sample-takers may also be well-placed to provide information at cervical screening appointments. However, healthcare professionals can have limitations to their own knowledge of HPV and may require different forms of training (McSherry et al., 2018, Mcsherry et al., 2012, Sherman et al., 2019, Tatar et al., 2019). Efforts have been made to develop healthcare professional training materials in England, but interventions may be required to facilitate learning outcomes and engagement (Public Health England, 2020). Best practice guidelines relating to the communication of cervical screening results are limited for those testing HPV+/normal both nationally and internationally. Most guidelines highlight covering generic information that HPV is common and can clear naturally, and when women should expect to attend their next screening appointment (Royal College of Nursing’s Women’s Health Forum Committee, 2020, Department of Health, 2017, Health Sevice Executive, 2019, Public Health Wales, 2019). Further work is needed to design tailored communication strategies for women testing HPV+/normal.

Visual aids or links to online videos were discussed and have been suggested as a potential solution to help women interpret complex information, particularly statistical information relating to cancer risk (Okan et al., 2019). A systematic review on cancer risk and screening information found that using behavioural science to convey personalisation can improve understanding of cancer and risk when compared with generic information (Albada et al., 2009). Implementing similar suggestions in HPV+/normal letters may aid interpretation and improve the experience of receiving results.

As HPV primary screening is implemented worldwide, our findings point to areas which can be used by policymakers and healthcare professionals to improve content and communication of HPV+/normal results. Pragmatic suggestions for letter restructuring paired with adoption of evidence-based content could help women process and better understand an HPV+/normal result. Our results were relatively homogenous by level of education; therefore, further quantitative research would be needed to ascertain whether differing educational materials would be needed for each group. As health systems move towards more digital approaches, this may provide opportunities for personalised screening services rather than a one-size-fits-all national programme. Since our study took place, the NHSCSP in England has incorporated a new frequently asked questions (FAQ) section as part of their HPV+/normal result letters, which should begin to address some of the concerns raised by women. Similar policy could be adopted in other countries. Topics and suggestions highlighted in this study can be used to cost-effectively develop, enhance, or refine FAQs and leaflet content in addition to improving letter wording. Targeted efforts to deliver healthcare professional training on HPV primary screening and communication skills may also facilitate provision of information to women at screening.

### 9.1 Strengths and limitations

To our knowledge, this is the first qualitative study to explore information needs in women receiving routine HPV+/normal results at HPV primary screening. Ecological validity was ensured through recruitment linked to routine clinical management at HPV primary screening. Our relatively diverse and well‐characterised sample allowed us to draw thematic comparisons between low- and high-education groups, which may be important for identifying inequalities in burden. However, our sample did not represent women without formal qualifications, who may represent a distinct group in terms of health literacy and need. Due to the relatively small numbers, we were unable to explore intersections between other important sociodemographic factors, such as ethnicity or age. Recall bias could be a limitation given interviews were carried out on average 35.5 days after result. Lastly, the primary qualitative study used for our secondary analysis purposively sampled women based on varying anxiety scores, which could be considered both a strength and limitation. Nonetheless, it is important that our secondary findings are interpreted in this context.

## 10 Conclusion

Women across all levels of education had unanswered questions about their HPV-positive with normal cytology result and the HPV primary screening protocol. Given that HPV primary screening is still a novel programme that continues to be piloted and implemented internationally, this research provides timely suggestions for service improvement whilst advancing the academic literature. Future research should use the suggestions outlined in this paper to develop and test patient communications with women from a range of sociodemographic backgrounds; and assess content engagement and information recall.

## Funding

This study was funded by the 10.13039/501100000272National Institute for Health Research (NIHR) as part of a fellowship awarded to Emily McBride (DRF-2017-10-105); the views expressed in this paper are not necessarily those of the NHS, the NIHR, or the Department of Health and Social Care. Jo Waller was funded by 10.13039/501100000289Cancer Research UK (C7492/A17219).

## CRediT authorship contribution statement

Emily McBride conceived the study, managed the project, and conducted the participant interviews. Sophie Mulcahy Symmons and Emily McBride analysed the data and drafted the manuscript. All authors contributed to, and approved, the final version of the manuscript.

## Declaration of Competing Interest

The authors declare the following financial interests/personal relationships which may be considered as potential competing interests: Dr Emily McBride and Dr Jo Waller sat on the NHS Cervical Screening Programme, HPV Primary Screening Pilot Steering Committee from 2016 to 2019.
