# Supplementary material for: Testing positive for Human Papillomavirus (HPV) at primary HPV cervical screening: A qualitative exploration of women’s information needs and preferences for communication of results
Source: Prev Med Rep. 2021 Aug 19;24:101529. doi: 10.1016/j.pmedr.2021.101529 (PMC8683976; doi:10.1016/j.pmedr.2021.101529)
Supplement: Supplementary Data 1 [file mmc1.docx]

**Supplementary File 1** – Wording in the HPV-positive with normal cytology result letter used through the English HPV primary cervical screening pilot.

##

Dear <<NAME>>

Thank you for coming for NHS cervical screening.

Your screening sample was tested for the human papillomavirus (HPV), and evidence of the virus was found. This is called an ‘HPV positive’ result.

Your screening sample was also tested for abnormal cervical cells. This test is called ‘cytology’. The results were normal (no abnormal cells were found).

Because HPV was found in your sample, we would like you to come back for screening again sooner than usual. This is so that we can check that the HPV has been cleared by your immune system (like getting rid of a cold).

Your next screening test is due on or around <<PAT_REC_DATE>>. We will send you a reminder letter nearer the time.

Cervical screening, like other medical tests, isn’t perfect. If you have any unusual symptoms such as a discharge, or bleeding between periods or after sex, then please speak to a GP. Cervical screening is not a test for symptoms.

If you have any questions about your test result or would like more information about cervical screening or HPV testing, please contact a GP or the person who did your last test.
